# Cerebellar Hemorrhage in Preterm Infants: A Meta-Analysis on Risk Factors and Neurodevelopmental Outcome

**DOI:** 10.3389/fphys.2019.00800

**Published:** 2019-06-25

**Authors:** Eduardo Villamor-Martinez, Monica Fumagalli, Yaser Ibrahim Alomar, Sofia Passera, Giacomo Cavallaro, Fabio Mosca, Eduardo Villamor

**Affiliations:** ^1^Department of Pediatrics, School for Oncology and Developmental Biology (GROW), Maastricht University Medical Center, Maastricht, Netherlands; ^2^Neonatal Intensive Care Unit, Fondazione IRCCS Cà Granda Ospedale Maggiore Policlinico, Milan, Italy; ^3^Department of Clinical Sciences and Community Health, University of Milan, Milan, Italy

**Keywords:** cerebellar hemorrhage, prematurity, meta-analysis, systematic review, risk factors, neurodevelopmental outcome, cerebral palsy

## Abstract

Cerebellar hemorrhage (CBH) represents the most commonly acquired lesion of the posterior fossa in the neonatal period. We aimed to perform a systematic review and meta-analysis of studies exploring the perinatal risk factors and neurological outcome of CBH in preterm infants. A comprehensive literature search was conducted using PubMed/MEDLINE and EMBASE. Studies were included if they examined preterm infants and reported primary data on maternal, obstetric, or perinatal characteristics, and/or outcomes of infants with and without CBH. A random-effects model was used to calculate mean differences (MD), odds ratios (OR), and 95% confidence intervals (CI). We found 231 potentially relevant studies, of which 15 met the inclusion criteria (4,236 infants, 347 CBH cases). Meta-analysis could not demonstrate a significant association between CBH and multiple gestation, chorioamnionitis, pre-eclampsia, placental abruption, use of antenatal corticosteroids, mode of delivery, or infant sex. Infants with CBH had a significantly lower gestational age (6 studies, MD −1.55 weeks, 95% CI −1.93 to −1.16) and birth weight (6 studies, MD −173 g, 95% CI −225 to −120), and significantly higher rates of intubation at birth, hypotension, patent ductus arteriosus, intraventricular hemorrhage, sepsis, necrotizing enterocolitis, and bronchopulmonary dysplasia. CBH was significantly associated with delayed mental (6 studies, OR 2.95, 95% CI 1.21 to 7.20) and psychomotor (6 studies, OR 3.62, 95% CI 1.34 to 9.76) development, and higher rates of cerebral palsy (4 studies, OR 3.09, 95% CI 1.55 to 6.19). In conclusion, the present meta-analysis shows that the youngest and sickest preterm infants are at higher risk of developing CBH. Our results highlight the multifactorial nature of CBH and reinforce the idea that cerebellar injury in very preterm newborns has important neurodevelopmental consequences among survivors.

## Introduction

In the last decade, imaging of the posterior fossa in preterm infants has gained increased attention. This has been due to the growing awareness of the occurrence of cerebellar injury (Volpe, 2009; Limperopoulos et al., 2018) and the recent evidence highlighting the impact of perinatal acquired cerebellar lesions on a wide spectrum of cerebral functions including cognition, language, and memory (Brossard-Racine et al., 2015; Hortensius et al., 2018). Cerebellar hemorrhage (CBH) represents the most commonly acquired lesion of the posterior fossa in the neonatal period with the youngest infants being at the highest risk (Volpe, 2009; Brossard-Racine et al., 2015; Fumagalli et al., 2015; Hortensius et al., 2018; Limperopoulos et al., 2018).

During the third trimester of pregnancy, the cerebellum undergoes a rapid and precisely programmed growth resulting in a 3.5-fold increase in volume and a 30-fold increase in surface area (Limperopoulos et al., 2005b; Volpe, 2009; Du Plessis et al., 2018). This growth is mainly related to the development of the external granule cell layer (Dobbing, 1974; Volpe, 2009). Between 24 and 30 weeks' gestation a friable capillary network with poor supportive stroma is observed in the germinal matrix of the fourth ventricle and in the subpial region of the external granular layer. These structures are very vulnerable to perfusion–reperfusion injury and have been accounted as a possible source of CBH. More recently, the potential role of internal granule cell layer has also been highlighted, considering its dense vascularization and cellularity (Pierson and Al Sufiani, 2016).

The above-described critical phase of development renders the cerebellum very vulnerable, especially when preterm birth induces early exposure to the extrauterine environment (Limperopoulos et al., 2005a; Volpe, 2009). Indeed, the transitory presence of immature vasculature in a context of impaired cerebral autoregulation and hemodynamic disturbances, such as fluctuations in venous pressure, renders very, and extremely preterm infants highly susceptible to develop CBHs. Moreover, the well-documented association of CBH with supratentorial germinal matrix–intraventricular hemorrhage (GM-IVH), suggests possible common risk factors and pathogenetic mechanisms (Fumagalli et al., 2015).

There is a broad spectrum in the severity of CBHs reported, ranging from mild punctate lesions, focal unilateral lesions, to the less common and more extensive bihemispheric and vermian hemorrhages (Parodi et al., 2015; Limperopoulos et al., 2018). Recently, a grading scheme was proposed: grade 1 consisted of unilateral small ( ≤ 3 mm) punctate lesions; grade 2 consisted of bilateral small punctate lesions; grade 3 consisted of extensive (>3 mm) unilateral lesions; and grade 4 consisted of extensive bilateral lesions (Neubauer et al., 2017). The addition of the mastoid fontanel window in cranial ultrasound (cUS) improved the detection of CBH compared to detection by the anterior and posterior fontanel cUS views alone (Limperopoulos et al., 2018). However, the majority of punctate hemorrhages may remain undetected, even when the mastoid fontanel approach is used (Steggerda et al., 2009; Steggerda and Van Wezel-Meijler, 2016). These small lesions are only detected by magnetic resonance imaging (MRI) scan, in particular on Susceptibility Weighted Imaging (SWI) sequence (Steggerda et al., 2009; Parodi et al., 2015; Steggerda and Van Wezel-Meijler, 2016; Limperopoulos et al., 2018). The reported incidence of focal CBHs detected by cUS ranged from 3.7 % in infants below 30 weeks of gestation (Sehgal et al., 2009) to 9% in infants below 32 weeks of gestation, when using the mastoid fontanel windows (Steggerda et al., 2009), but increased to 19% when MRI was used (Steggerda et al., 2009). Long-term neurodevelopmental prognosis of CBH is related to the location and extension of the lesion and, although still debated, growing evidence shows a wide range of neurodevelopmental impairments, including high-order cerebral functions (Volpe, 2009; Brossard-Racine et al., 2015; Hortensius et al., 2018; Limperopoulos et al., 2018).

Etiopathogenetic mechanisms may differ between large and small CBHs but it is generally accepted that the incidence of CBH is strikingly dependent on the degree of prematurity (Volpe, 2009; Limperopoulos et al., 2018). Nevertheless, very and extremely preterm infants have specific perinatal risk factors and some of these factors, such as lack of antenatal steroids, intubation at birth, patent ductus arteriosus (PDA), need for inotropic drugs acidosis during the first days of life, or sepsis, have been reported to confer additional risk for the development of CBH (Limperopoulos et al., 2005a, 2018; Sehgal et al., 2009; Volpe, 2009; Mccarthy et al., 2011; Chau et al., 2012; Zayek et al., 2012; Neubauer et al., 2017). However, the evidence is still unclear mainly due to the heterogeneity of the studied populations, the small sample sizes, and the different risk factors analyzed. A more accurate identification of risk factors for CBH would help in defining infants at high risk who may deserve in-depth neuroimaging investigation of posterior fossa and who may benefit from early intervention strategies. Therefore, we aimed to perform a systematic review and meta-analysis of studies exploring the perinatal risk factors and neurological outcomes of CBH in preterm infants.

## Methods

The study was conducted according to the Preferred Reporting Items for Systematic Reviews and Meta-Analysis (PRISMA) (Moher et al., 2009). A protocol was developed prospectively that detailed the specific objectives, criteria for study selection, the approach to assessing study quality, clinical outcomes, and statistical methodology. The study is reported according to the PRISMA checklist (see Supplementary Material).

### Sources and Search Strategy

We searched PubMED (MEDLINE) and EMBASE for relevant studies. We searched from the inception of the databases until May 2017. The strategy for EMBASE was “exp cerebellum hemorrhage/ AND exp prematurity/,” in addition to related free text terms (“cerebellar hemorrhage,” “cerebellum hemorrhage,” “cerebellar hemorrhage,” “pre-mature infant,” “pre-term baby,” “pre-term child,” “pre-term infant,” “pre-term neonate,” “pre-term newborn,” “premature,” “prematuritas,” “preterm baby,” “preterm child,” “preterm infant,” “preterm neonate,” “preterm newborn”). We used a similar strategy for PubMED, with the MeSH terms for “cerebellar hemorrhage” and “neonatal prematurity” in addition to related free text terms. We reviewed reference lists of relevant articles and reviews for additional studies. We did not exclude studies based on language, and studies were translated where necessary.

### Study Selection

We included studies that compared infants with CBH to infants without CBH and reported on risk factors for and/or outcomes of CBH. We included studies which only included preterm (<37 weeks) and low birth weight (<2,500 g) infants. Studies without data on a control group were excluded. Two reviewers (EV-M, YA) screened studies for inclusion independently on title and abstract. In case of disagreement, the study was included and reevaluated in the second round of inclusion. The second round of inclusion was based on full-text screening, and discrepancies between reviewers were resolved through discussion or in consultation with a third reviewer (EV).

### Data Extraction

Using a predefined worksheet, one researcher (YA) extracted data from included studies. We extracted the following data from each study: citation information, location of study, study period, primary objective, criteria for inclusion/exclusion of infants, definitions used for CBH, baseline characteristics, risk factors and outcomes (including raw numbers, summary statistics and adjusted analyses when available). Two researchers (SP, EV-M.) checked extracted data for accuracy and completeness. We resolved discrepancies through discussion and by consulting the primary report.

### Quality Assessment

We used the Newcastle-Ottawa Scale (NOS) for cohort or case control studies to assess the methodological quality of included studies (Wells et al., 2001). This scale uses a rating system (range: 0–9 points) that scores three aspects of a study: selection of the study groups (0–4 points), comparability of the study groups (0–2 points) and ascertainment of exposure/outcome (0–3 points) (Wells et al., 2001). Two researchers (EV-M. and EV) independently used the NOS to evaluate the quality of each study, and discrepancies were discussed and resolved by consensus.

### Statistical Analysis

We combined and analyzed studies using comprehensive meta-analysis V 3.0 software (CMA, RRID:SCR_012779, Biostat Inc., Englewood, NJ, USA). We calculated the odds ratio (OR) and 95% confidence intervals (CI) for dichotomous outcomes from the data extracted from the studies. We calculated the mean difference (MD) and 95% CI for continuous outcomes. We used the method of Wan and colleagues (Wan et al., 2014) to estimate the mean and standard deviation when continuous variables were reported as median and range/interquartile range in studies. We used a random-effects model to calculate summary statistics, due to anticipated heterogeneity. This method accounts for both intra-study and inter-study variability. We considered a probability value below 0.05 as statistically significant (0.10 for heterogeneity). We carried out publication bias analyses for outcomes reported in at least 15 studies (visual inspection of the funnel plot and Egger's regression test).

## Results

### Description of Studies

**Figure 1:**
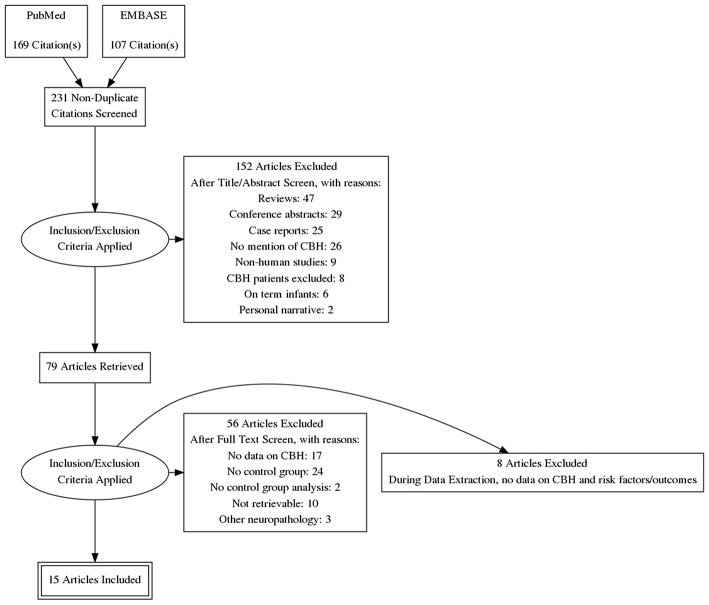
PRISMA flow diagram of the systematic search.

Of 231 potentially relevant studies, 15 met the inclusion criteria (Limperopoulos et al., 2005a, 2007; Dyet et al., 2006; O'Shea et al., 2008; Fumagalli et al., 2009; Biran et al., 2011; Tam et al., 2011; Chau et al., 2012; Zayek et al., 2012; Duerden et al., 2013; Haines et al., 2013; Steggerda et al., 2013; Kidokoro et al., 2014; Gano et al., 2016; Neubauer et al., 2017). The PRISMA flow diagram of the search is shown in Figure 1. The included studies evaluated 4,236 infants, including 347 cases of CBH. The included studies and their characteristics are summarized in Table 1. Of the 15 studies, one (Steggerda et al., 2013) analyzed cerebellar punctate lesions, 7 analyzed focal lesions, 5 analyzed all type of lesions, and 2 did not clarify which types of CBH lesions they studied (Table 1). Two studies included infants with GA<28 weeks (O'Shea et al., 2008; Zayek et al., 2012), 3 studies included infants with GA<30 weeks (Dyet et al., 2006; Biran et al., 2011; Kidokoro et al., 2014), 5 studies included infants with GA<32 weeks (Limperopoulos et al., 2007; Chau et al., 2012; Duerden et al., 2013; Steggerda et al., 2013; Neubauer et al., 2017), 2 studies included infants with GA<33weeks (Fumagalli et al., 2009; Gano et al., 2016), one study included infants with GA<34weeks (Tam et al., 2011), and 2 studies included infants with GA<37weeks (Limperopoulos et al., 2005a; Haines et al., 2013).

**Table 1:** Synoptic table of study characteristics.

**Study**	**Study type**	**Included infants (centers)**	**Max GA**	**Max BW**	**Method of CBH diagnosis**	**CBH lesions studied**
Biran et al., 2011	Retrospective case-control	30 (1)	29 6/7	N/A	MRI	Focal lesions
Chau et al., 2012	Prospective cohort	117 (1)	32	N/A	MRI	Unclear
Duerden et al., 2013	Prospective cohort	133 (1)	32	N/A	MRI	Unclear
Dyet et al., 2006	Prospective cohort	119 (1)	29 6/7	N/A	MRI	Focal lesions
Fumagalli et al., 2009	Prospective cohort	75 (1)	33	1000	MRI	Punctate and focal lesions
Gano et al., 2016	Prospective cohort	73 (1)	32 6/7	N/A	MRI	Punctate and focal lesions
Haines et al., 2013	Retrospective cohort	45 (1)	36 6/7	N/A	Autopsy	Focal lesions
Kidokoro et al., 2014	Prospective cohort	325 (3)	29 6/7	N/A	MRI	Punctate and focal lesions
Limperopoulos et al., 2005a	Retrospective case-control	105 (1)	36 6/7	N/A	cUS, mastoid view	Focal lesions
Limperopoulos et al., 2007	Retrospective case-control	86 (2)	31 6/7	N/A	cUS, mastoid view and MRI in early childhood	Focal lesions
Neubauer et al., 2017	Retrospective cohort	300 (1)	31 6/7	N/A	MRI	Punctate and focal lesions
O'Shea et al., 2008	Prospective cohort	1445 (14)	27 6/7	N/A	cUS, anterior fontanel	Focal lesions
Steggerda et al., 2013	Prospective cohort	132 (1)	31 6/7	N/A	MRI scan and cUS	Punctate lesions
Tam et al., 2011	Prospective cohort	131 (1)	33 6/7	N/A	MRI, and cranial ultrasound	Punctate and focal lesions
Zayek et al., 2012	Retrospective cohort	1120 (1)	27 6/7	N/A	cUS, anterior and posterior fontanels	Focal lesions

*GA, gestational age; BW, birth weight; CBH, cerebellar hemorrhage; MRI, magnetic resonant imaging; cUS, cranial ultrasound*.

### Quality Assessment and Publication Bias

The quality assessment for each study according to the NOS is shown in Supplementary Table 1. Studies were downgraded in quality for not adjusting/matching for confounders (*k* = 7) and for not clearly defining CBH (*k* = 3). We did not carry out an analysis of publication bias for any of the included outcomes due to the low number of studies per outcome (*k* < 15).

### Meta-Analyses on Demographic Characteristics and Maternal and Obstetric Risk Factors

**Figure 2:**
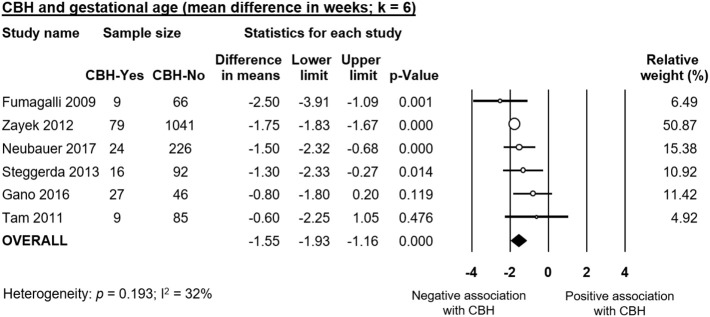
Meta-analysis of mean difference in gestational age, comparing cerebellar hemorrhage (CBH)-group and control group.

**Figure 3:**
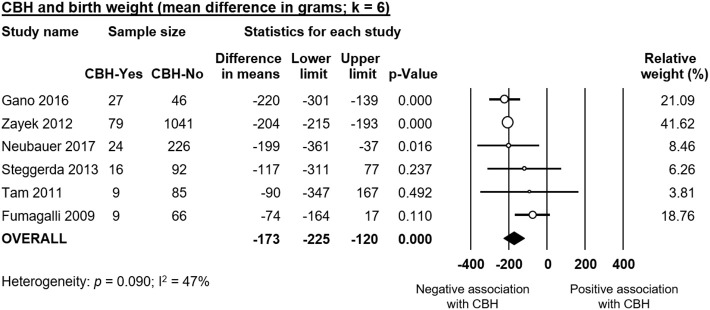
Meta-analysis of mean difference in birth weight, comparing cerebellar hemorrhage (CBH)-group and control group.

**Figure 4:**
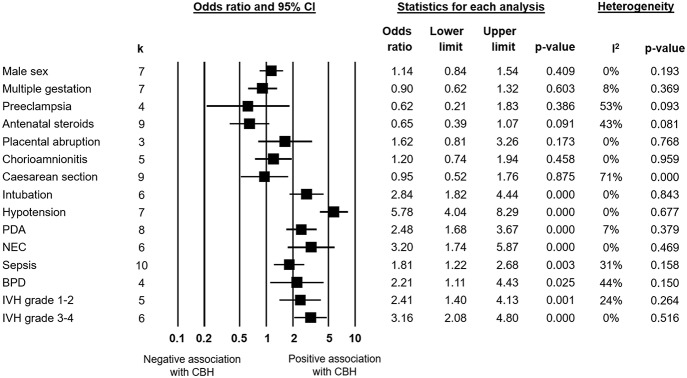
Meta-analysis of the association between cerebellar hemorrhage (CBH) and maternal, obstetric and perinatal characteristics, and short-term neonatal outcomes. PDA, patent ductus arteriosus; NEC, necrotizing enterocolitis; BPD, bronchopulmonary dysplasia; IVH, intraventricular hemorrhage.

Infants with CBH had significantly lower GA (MD in weeks −1.55, 95% CI −1.93 to −1.16, Figure 2) and significantly lower BW (MD in g −173, 95% CI −225 to −120, Figure 3). In contrast, meta-analysis could not find a significant association between CBH and infant sex (Figure 4 and Supplementary Figure 1), multiple gestation (Figure 4 and Supplementary Figure 2), preeclampsia (Figure 4 and Supplementary Figure 3), use of antenatal steroids (Figure 4 and Supplementary Figure 4), placental abruption (Figure 4 and Supplementary Figure 5), chorioamnionitis (Figure 4 and Supplementary Figure 6), or cesarean section (Figure 4 and Supplementary Figure 7). In addition, maternal age was not significantly different between the CBH and the control group (3 studies, MD −0.44, 95% CI −2.51 to 1.63, Supplementary Figure 8).

### Clinical Conditions and Short-Term Outcomes

We found in meta-analysis that CBH had a positive association with intubation at birth (Figure 4 and Supplementary Figure 9), hypotension (Figure 4 and Supplementary Figure 10), patent ductus arteriosus (Figure 4 and Supplementary Figure 11), necrotizing enterocolitis (NEC, Figure 4 and Supplementary Figure 12), sepsis (Figure 4 and Supplementary Figure 13), bronchopulmonary dysplasia (BPD, Figure 4 and Supplementary Figure 14), grade 1–2 IVH (Figure 4 and Supplementary Figure 15), and grade 3-4 IVH (Figure 4 and Supplementary Figure 16). CBH was also significantly associated with a lower pH in the first week of life, though only one study reported on this outcome (Limperopoulos et al., 2005a). Meta-analysis on Apgar score could not be performed due to the heterogeneity of reported data but two studies (Limperopoulos et al., 2005a; Neubauer et al., 2017) reported significant lower Apgar scores in infants with CBH.

### Neurodevelopmental Outcome

**Figure 5:**
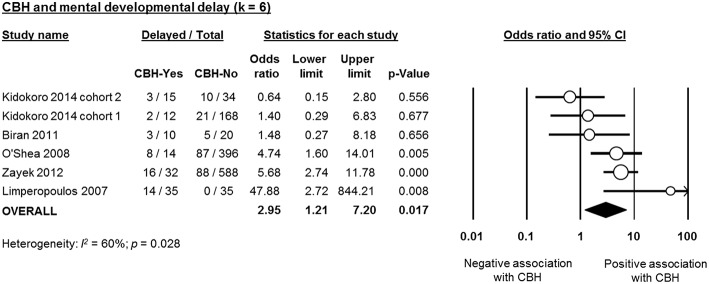
Meta-analysis of the association between cerebellar hemorrhage (CBH) and risk of delayed mental development. CI, confidence interval.

**Figure 6:**
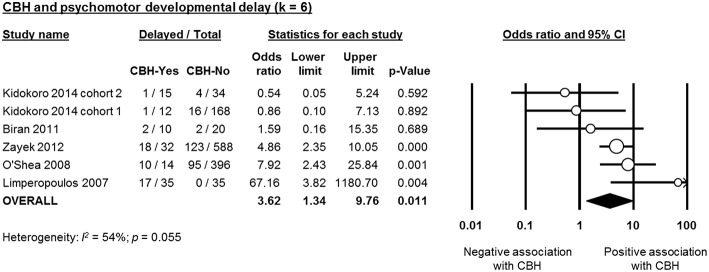
Meta-analysis of the association between cerebellar hemorrhage (CBH) and risk of delayed psychomotor development. CI, confidence interval.

**Figure 7:**
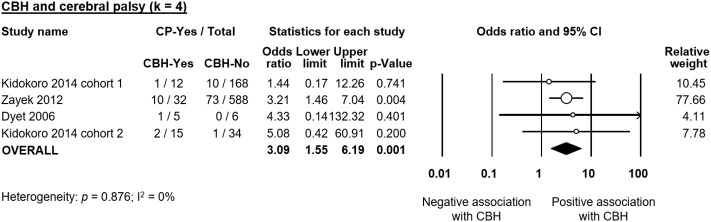
Meta-analysis of the association between cerebellar hemorrhage (CBH) and risk of cerebral palsy (CP). CI, confidence interval.

Six studies reported data on neurodevelopmental outcome of CBH-exposed and control infants. The assessment methods, timing and summary of findings are summarized in Supplementary Table 2. We found through meta-analysis that CBH was significantly associated with delayed mental (6 studies, OR 2.95, 95% CI 1.21 to 7.20, Figure 5) and psychomotor (6 studies, OR 3.62, 95% CI 1.34 to 9.76, Figure 6) development, and higher rates of cerebral palsy (4 studies, OR 3.09, 95% CI 1.55 to 6.19, Figure 7).

## Discussion

To the best of our knowledge, this is the first meta-analysis providing a comprehensive estimation of antenatal, perinatal, and early postnatal risk factors associated with the development of CBH in preterm infants. The current meta-analysis strengthens and quantifies the previous concept of the multifactorial etiopathogenesis of CBH highlighting the central role of preterm birth and its related complications, while the relevance of maternal and obstetric factors remains unclear. Infants with CBH were born earlier, they had a lower BW, and they were more frequently exposed to systemic hypotension, PDA, low (1–2) and high (3–4) grade IVH, NEC, sepsis, and BPD. In addition, our meta-analysis confirms the findings of previous systematic reviews (Brossard-Racine et al., 2015; Hortensius et al., 2018) that showed that the presence of CBH is associated with a higher risk of impaired neurodevelopment in preterm infants. However, the presence of undiagnosed minor brain abnormalities or impaired brain growth associated with preterm birth may partially account for the long-term neurodevelopmental disorders observed in preterm infants with CBH.

The higher incidence of both large (Limperopoulos et al., 2005a; Gano et al., 2016) and small CBHs (Steggerda et al., 2013) in infants with lower GA and BW may be related to the anatomic characteristics of the developing cerebellum. In particular, the immature vessels of the preterm cerebellum are more sensitive to hemodynamic disturbances (Pierson and Al Sufiani, 2016). The central role of hemodynamic disturbances (hypotension and PDA) in the context of an extreme immature anatomic and functional brain structure is supported by the timing of occurrence of CBH, with usually in the first days of life, when preterm infants experienced a critical period in terms of hemodynamic and respiratory adaptation. Moreover, agents that reduce fluctuations in blood pressure, as magnesium sulfate, have been demonstrated to exert a neuroprotective effect (Gano et al., 2016), while interventions, like high dose caffeine, (Mcpherson et al., 2015), or surgery (Stolwijk et al., 2017), which are likely to impact on systemic hemodynamics are associated with a higher risk of developing CBH.

Among preterm infants, males appear to have a higher incidence and increased severity of brain lesions, such as IVH and periventricular leukomalacia (PVL) (Mohamed and Aly, 2010; O'Driscoll et al., 2018). It has been suggested that intrauterine differences in hormonal environment (Nuñez and Mccarthy, 2003) as well as the higher cerebral blood flow present in preterm male infants (Baenziger et al., 1994) are responsible for the higher susceptibility of preterm male neonates to brain injury (O'Driscoll et al., 2018). In contrast, in the present study, neither the individual studies (Supplementary Figure 1) nor the meta-analysis results suggested sex differences in the incidence of CBH.

### Maternal and Obstetric Factors

Considering the multifactorial etiology of CBH, we analyzed the contribution of maternal and obstetric factors (Figure 4). Meta-analysis could not find that any of these factors, including maternal age, chorioamnionitis, preeclampsia, multiple pregnancy, mode of delivery, or use of antenatal corticosteroids is significantly associated with the development of CBH.

Chorioamnionitis is a well-known risk factor for preterm birth and prematurity-associated morbidities due to the activation of the inflammatory pathways (Hartling et al., 2012; Mitra et al., 2014; Behbodi et al., 2016; Villamor-Martinez et al., 2018a,b). The role of intrauterine inflammation is well-known as an underlying pathogenetic mechanism of white matter disease of prematurity (Strunk et al., 2014). Very recently, we showed in a meta-analysis that both clinical and histological chorioamnionitis were associated with an increased risk for developing IVH in very preterm infants (Villamor-Martinez et al., 2018b). Interestingly, and in contrast to other complications of prematurity, such as PDA, ROP, or BPD (Hartling et al., 2012; Mitra et al., 2014; Behbodi et al., 2016; Villamor-Martinez et al., 2018a), the effect of chorioamnionitis on IVH appeared to be independent of chorioamnionitis as a causative factor of prematurity. Considering that IVH and CBH share common pathogenetic mechanisms, we expected to see an effect of chorioamnionitis on the risk of developing CBH but this was not the case. A small number of studies reporting on chorioamnionitis, and the heterogeneity of the inclusion criteria for chorioamnionitis, varying from clinical (Kidokoro et al., 2014; Gano et al., 2016) to histological (Limperopoulos et al., 2005a) or not specified (Sehgal et al., 2009; Haines et al., 2013 may account for this discrepancy.

Only four studies included in meta-analysis reported data on pre-eclampsia (Limperopoulos et al., 2005a; Fumagalli et al., 2009; Haines et al., 2013; Gano et al., 2016) and all of them observed a trend, albeit a non-significant one, toward reduction of CBH in infants born from mothers with pre-eclampsia. Recently, pre-eclampsia has been suggested as a protective factor for supratentorial bleeding in very (Morsing et al., 2018) and extremely (Gemmell et al., 2016) preterm infants. However, evidence is still controversial considering the potential role of many confounding factors such as the higher likelihood of planned delivery, cesarean section, and of receiving antenatal steroids, alongside greater prenatal surveillance of mothers with pre-eclampsia. Of note, although cesarean section is suggested to protect against IVH (Inder et al., 2018), our meta-analysis could not find a similar protective effect against CBH.

One important limitation in interpreting our negative results on the association between multiple gestation and CBH is the lack of data on chorionicity. Monochorionic pregnancy are likely to be complicated by placental hemodynamic alterations which may result in fetal hemodynamic disturbances (as twin-to-twin-transfusion) playing a role in the pathogenesis of CBH.

Maternal administration of corticosteroids in case of anticipated preterm delivery reduces neonatal mortality and morbidity, including IVH (Handley et al., 2018), and has become standard of care in current obstetric practice (Miracle et al., 2008). Many mechanisms have been suggested for the protective effects of antenatal corticosteroids, among which the induction of lung maturation and the reduction of severity of postnatal respiratory disease (Xu et al., 2008; Vinukonda et al., 2010; Wapner, 2013). However, and although one of the included studies suggested a protective effect of antenatal corticosteroids on CBH development (Neubauer et al., 2017), meta-analysis could not demonstrate a significant effect (Figure 4). Nevertheless, due to the low robustness of the results, the inclusion of future studies may produce relevant changes in the effect size of the association between antenatal corticosteroids and CBH.

### Clinical Conditions and Short-Term Outcomes

Our analysis confirms that the need of resuscitation with intubation at birth represents a risk factor for CBH. Meta-analysis on Apgar score could not be performed due to the heterogeneity of reported data but two studies (Limperopoulos et al., 2005a; Neubauer et al., 2017) reported significant lower Apgar scores in infants with CBH. Major early postnatal complications were significantly associated with CBH confirming that the most immature and sickest infants, who suffer the most severe morbidities, are the ones more prone to develop CBH (Sehgal et al., 2009; Neubauer et al., 2017). Sepsis, systemic hypotension, PDA and acidosis in the first 7 days increase the risk of CBH. All these factors, directly or indirectly, induce fluctuations in cerebral blood flow which in turn increase the risk of bleeding. These factors play a role in the complex interplay of pathogenetic mechanisms of CBH but unfortunately, we were not able to assess their independent role and to disentangle the potential effect of prematurity itself.

The association between CBH and supratentorial IVH is well-documented (Fumagalli et al., 2015) suggesting common pathogenetic mechanisms. Indeed, the germinal matrix, which is supposed to be the origin of both IVH and CBH, is present both in the cerebellum and supratentorially (Volpe, 2009; Limperopoulos et al., 2018). Moreover, large CBH can also occur secondary to extension of intraventricular or sub- arachnoid blood into the cerebellum as a consequence of a dissection of blood through the fourth ventricle or subarachnoid spaces following massive IVH (Limperopoulos et al., 2008). Similarly, Parodi et al. speculated that cerebellar microhemorrhages, which are mostly located in the external portion of cerebellar parenchyma, might represent haemosiderin depositions originating from a supratentorial bleeding and conveyed by the cerebrospinal fluid to the subarachnoid space of the posterior fossa (Parodi et al., 2015).

### Neurodevelopmental Outcome

As mentioned in the introduction, the systematic review of Hortensius et al. showed a high incidence of severe neurodevelopmental impairment in preterm infants with isolated CBH in the cognitive, motor, language, and behavioral domains (Hortensius et al., 2018). Our meta-analysis confirmed that the presence of CBH in preterm infants increased the risk of impaired mental and psychomotor development as well as the rate of CP. However, our results are limited by the low number of studies and the marked heterogeneity that did not allow us to investigate the effect of the location (unilateral, bilateral, or with vermis involvement) and the size (punctate or large) of CBH on neurodevelopmental outcome. Vermis involvement and large CBHs have been related with higher rates of neurodevelopmental impairment (Hortensius et al., 2018).

In addition, some of the short-term complications that are significantly more frequent in infants with CBH, such as sepsis, NEC, or BPD may have a marked, CBH-independent, influence on neurodevelopmental outcome through both additive and interactive roles (Hortensius et al., 2018). Moreover, the studies used different follow-up periods and different scales, or different editions of the Bayley scale, for the assessment of neurodevelopmental outcome (Supplementary Table 2) and the correlation between the cut-off points of the different scales to define neurodevelopmental delay is controversial (Moore et al., 2012; Johnson et al., 2014).

### Limitations

Our meta-analysis has some important limitations that hamper the practical application of the results. Firstly, and as mentioned above, we were only able to include a limited number of studies (*k* = 15), and studies reported on different risk factors and outcomes. Consequently, we probably lacked the power to find some associations. Secondly, studies showed heterogeneity in inclusion criteria, as well as in definition and assessment of CBH, risk factors, and outcomes, and 6 studies out of 15 did not control for confounding factors. Some of the included studies did not have the investigation of CBH as primary aim. In addition, it should be noted that only 2 studies exclusively included extremely preterm infants (GA < 28 weeks) (O'Shea et al., 2008; Zayek et al., 2012) and 2 studies included all preterm infants (GA < 37 weeks) (Limperopoulos et al., 2005a; Haines et al., 2013). The limited number of studies did not make it possible to stratify studies in subgroups to analyze the potential sources of heterogeneity, and we could not adjust for confounders through meta-regression. Finally, we did not define baseline characteristics and outcomes to include and analyze a priori but, due to the limited number of studies, decided on a case-by-case basis whether there was enough homogeneity to carry out meta-analysis, which has some potential to introduce bias.

## Concluding Remarks

The present meta-analysis highlights the multifactorial nature of CBH and reinforces the idea that cerebellar injury in very preterm newborns has important neurodevelopmental consequences among survivors. However, further research is warranted to understand the complex relationship between hemorrhagic cerebellar injury and its influence on neurodevelopmental outcome (Brossard-Racine et al., 2015; Hortensius et al., 2018). The cerebellum does not participate as an isolated entity in the integration of neural information, since the cerebral cortex and cerebellum of the mature brain are connected by a myriad, closed loop circuit, with afferent and efferent limbs, forming distinct functional, and structural units (Volpe, 2009; Limperopoulos et al., 2014). In very and extremely preterm infants, cerebellar injury occurs prior to maturation of cerebello-cerebral connectivity and, therefore, the remote effects of primary cerebellar injury may continue to influence cerebral development and plasticity over months to years (Volpe, 2009; Limperopoulos et al., 2014). Future studies should include large cohorts, with clear description of the topography and the size of CBHs, and other cerebellar and supratentorial injuries, and with risk factors and outcomes described on an individual patient level (Hortensius et al., 2018). In addition, research is needed in order to understand the relationship between prematurity-related cerebellar injury and other long-term outcomes such as autism spectrum disorders (Brossard-Racine et al., 2015).

## Author Contributions

EV-M selected studies for inclusion, carried out data collection, carried out statistical analyses, assessed methodological quality, contributed to interpretation of results, drafted part of the initial manuscript, and reviewed and revised the manuscript. MF contributed to the design of the study, the statistical analysis and interpretation of results, supervised data collection, drafted part of the initial manuscript, and reviewed and revised the manuscript. YA selected studies for inclusion, carried out data collection, contributed to statistical analyses and interpretation of results, and reviewed and revised the manuscript. SP checked extracted data for accuracy and completeness, contributed to interpretation of results, and reviewed and revised the manuscript. GC contributed to interpretation of results and reviewed and revised the manuscript. FM contributed to interpretation of results and reviewed and revised the manuscript. EV conceptualized and designed the study, supervised the search and selection of studies, supervised data collection, assessed methodological quality, contributed to statistical analyses and interpretation of results, and reviewed and revised the manuscript. All authors approved the final manuscript as submitted.

### Conflict of Interest Statement

MF and FM co-authored one of the studies included in the review and provided additional data. The remaining authors declare that the research was conducted in the absence of any commercial or financial relationships that could be construed as a potential conflict of interest.
