# Supplementary material for: Cerebellar Hemorrhage in Preterm Infants: A Meta-Analysis on Risk Factors and Neurodevelopmental Outcome
Source: Front Physiol. 2019 Jun 25;10:800. doi: 10.3389/fphys.2019.00800 (PMC6603211; doi:10.3389/fphys.2019.00800)
Supplement: Supplementary file 1 [file Data_Sheet_1.pdf]

Supplementary Material

Cerebellar hemorrhage in preterm infants: a meta-analysis on risk factors and neurodevelopmental outcome

Eduardo Villamor-Martinez[1#], Monica Fumagalli[2,3#], Yaser Ibrahim Alomar[1], Sofia Passera[2], Giacomo Cavallaro[2], Fabio Mosca[2,3], Eduardo Villamor[1*]

[1]Department of Pediatrics, School for Oncology and Developmental Biology (GROW), Maastricht University Medical Center, Maastricht, Netherlands

[2]Neonatal Intensive Care Unit, Fondazione IRCCS Cà Granda Ospedale Maggiore Policlinico, Milan, Italy

[3]Department of Clinical Sciences and Community Health, University of Milan, Milan, Italy

[#] Contributed equally to this work

*** Correspondence:**
Eduardo Villamor
e.villamor@mumc.nl

1 Supplementary Tables

Supplementary Table 1. Quality assessment of included studies using the Newcastle Ottawa Scale.

First author, year	Selection	Comparability	Outcome/Exposure	Total	Reason for downgrade
Biran, 2011	4	1	3	8	Only matched for GA
Chau, 2012	3	0	3	6	CBH not defined, No adjustment for confounders
Duerden, 2013	3	0	3	6	CBH not defined, No adjustment for confounders
Dyet, 2006	4	0	3	7	No adjustment for confounders
Fumagalli, 2009	4	0	3	7	No adjustment for confounders
Gano, 2016	4	2	3	9	
Haines, 2013	3	2	3	8	Infants not comparable with other studies (all were autopsied)
Kidokoro, 2014	4	2	3	9	
Limperopoulos, 2005a	4	2	3	9	
Limperopoulos, 2007	4	2	3	9	
Neubauer, 2017	4	0	3	7	No adjustment for confounders
O'Shea, 2008	4	0	3	7	No adjustment for confounders
Steggerda, 2013	4	2	3	9	
Tam, 2011	4	2	3	9	
Zayek, 2012	4	2	3	9	

GA: gestational age; CBH: cerebellar hemorrhage.

Supplementary Table 2. Details on studies that reported neurodevelopmental outcomes

First author, year	Assessment method	Time of assessment	Impairment defined as	Summary of findings	Comments
Biran 2011	Neurological examination, Brunet-Lezine test for developmental quotient,	2 years (not corrected)	Developmental quotient score <70	No significant difference between groups in any element of developmental quotient (global, postural, coordination, language or sociability).	Results of neurological examination not reported.
Dyet 2006	Neurological examination, CP, Griffiths Mental Development Scales for overall DQ	18-36 months (corrected)	Developmental quotient score <70	CBH-infants had lower DQ (75 +- 24, n=5), compared with infants with normal MRI (112 +- 15, n=6). One infant with CBH had CP, compared to none in the control group.	
Kidokoro 2014	Neurological examination, CP assessment. BSID-II and BSID-III scale for MDI and PDI, CP assessment.	2 years (corrected)	BSID-II: MDI score <70, PDI score <70. BSID-III: cognition or language score <80, motor score <70	CBH was not significantly related to any adverse neurodevelopmental outcome	Two different cohorts: one cohort was evaluated with BSID-II, another cohort was evaluated with BSID-III
O'Shea 2008	Neurological examination, BSID-II scale for MDI and PDI.	2 years (corrected)	MDI score <70 PDI score <70	Children who had bilateral cerebellar hemorrhages were at highest risk of developmental delays	Results of neurological examination not reported.
Steggerda 2013	Neurological examination according to Hempel, CP assessment (GMFCS), Neurodevelopment, BSID-III	2 years (corrected)	Score 1 SD below normative mean	No relation between small CBH and mildly or severely abnormal neurodevelopment.	Study only examined small CBH

First author, year	Assessment method	Time of assessment	Impairment defined as	Summary of findings	Comments
Tam 2011	Neurological examination, WPPSI-III for developmental outcome	3-6 years (not corrected)	Not defined	Infants with CBH had increased odds (OR 5.0, 95% CI 1.1-23.1) of neurological abnormalities at ages 3-6 compared to non-CBH infants, after adjusting for GA, IVH and white matter injury. No significant differences in developmental outcome.	
Zayek 2012	CP assessment, BSID-II and BSID-III for MDI and PDI.	12-18 months (corrected)	MDI score <70, or any cognitive or language score <70. PDI score <70, or motor score <70	Infants with CBH had higher rates of mental and motor impairment, and higher cerebral palsy rates. After adjustment, CBH was still associated with mental and motor impairment, but not with cerebral palsy.	

BSID-II: Bayley Scales of Infant Development, Second Edition; MDI: Mental Development Index; PDI: Psychomotor Developmental Index; CBH: cerebellar hemorrhage; WPPSI-III: Wechsler Preschool Primary Scale of Intelligence; CP: Cerebral Palsy; GMFCS: Gross Motor Function Classification System; IVH: intraventricular hemorrhage; DQ: developmental quotient.

2 Supplementary Figures

CBH and male sex (k = 7)

Study name	CBH-Yes / Total: Male	CBH-Yes / Total: Female	Odds ratio	Lower limit	Upper limit	p-Value	Relative weight (%)
Gano 2016	12 / 36	15 / 37	0.73	0.28	1.90	0.524	10.28
Steggerda 2013	9 / 64	7 / 44	0.86	0.30	2.53	0.791	8.15
Zayek 2012	39 / 570	40 / 550	0.94	0.59	1.48	0.778	44.75
Fumagalli 2009	4 / 34	5 / 41	0.96	0.24	3.90	0.954	4.77
Kidokoro 2014			1.70	0.79	3.68	0.178	15.71
Neubauer 2017	16 / 128	8 / 122	2.04	0.84	4.95	0.117	11.88
Tam 2011	6 / 46	3 / 48	2.25	0.53	9.59	0.273	4.46
**OVERALL**			**1.14**	**0.84**	**1.54**	**0.409**	

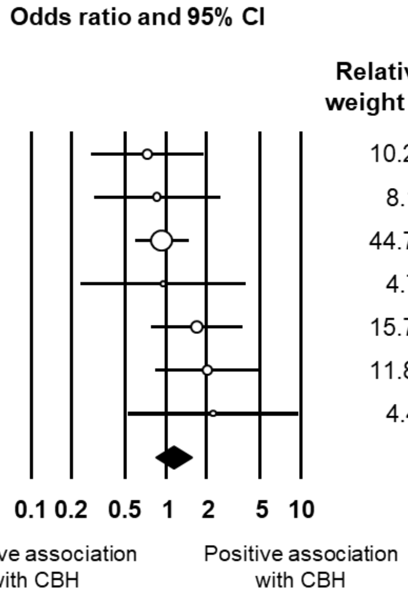

Heterogeneity: p = 0.193; I^2 = 32%

Supplementary Figure 1. Meta-analysis of cerebellar hemorrhage (CBH) and male sex. CI: confidence interval.

CBH and multiple gestation (k = 7)

Study name	Mult. Gest.-Yes / Total: CBH-Yes	Mult. Gest.-Yes / Total: CBH-No	Odds ratio	Lower limit	Upper limit	p-Value	Relative weight (%)
Biran 2011	0 / 10	5 / 20	0.13	0.01	2.69	0.189	1.56
Kidokoro 2014			0.55	0.23	1.31	0.176	16.97
Fumagalli 2009	1 / 9	12 / 66	0.56	0.06	4.93	0.603	2.95
Haines 2013	4 / 19	7 / 26	0.72	0.18	2.94	0.652	6.90
Gano 2016	11 / 27	22 / 46	0.75	0.29	1.96	0.557	14.04
Neubauer 2017	9 / 24	97 / 226	0.80	0.34	1.90	0.610	16.92
Zayek 2012	22 / 79	219 / 1041	1.45	0.87	2.42	0.158	40.66
**OVERALL**			**0.90**	**0.62**	**1.32**	**0.603**	

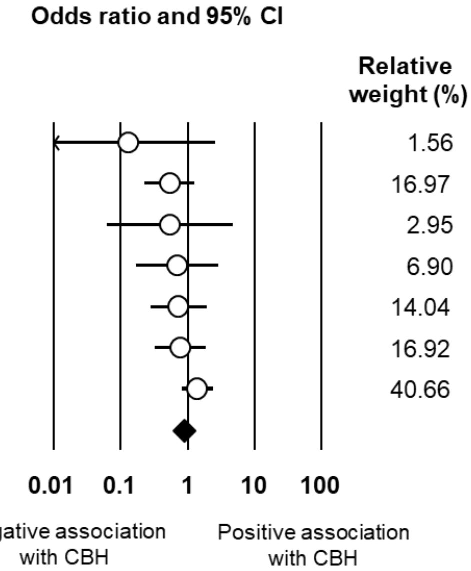

Heterogeneity: p = 0.369; I^2 = 8%

Supplementary Figure 2. Meta-analysis of cerebellar hemorrhage (CBH) and multiple gestation. CI: confidence interval.

CBH and preeclampsia (k = 4)

Study name	Preeclampsia-Yes / Total: CBH-Yes	CBH-No	Odds ratio	Lower limit	Upper limit	p-Value	Relative weight (%)
Fumagalli 2009	2 / 9	45 / 66	0.13	0.03	0.70	0.017	22.72
Gano 2016	5 / 27	12 / 46	0.64	0.20	2.08	0.462	30.94
Haines 2013	1 / 19	2 / 26	0.67	0.06	7.94	0.748	13.66
Limperopoulos 2005a	7 / 35	9 / 70	1.69	0.57	5.01	0.340	32.68
**OVERALL**			**0.62**	**0.21**	**1.83**	**0.386**	

Heterogeneity: *p* = 0.093; I^2 = 53%

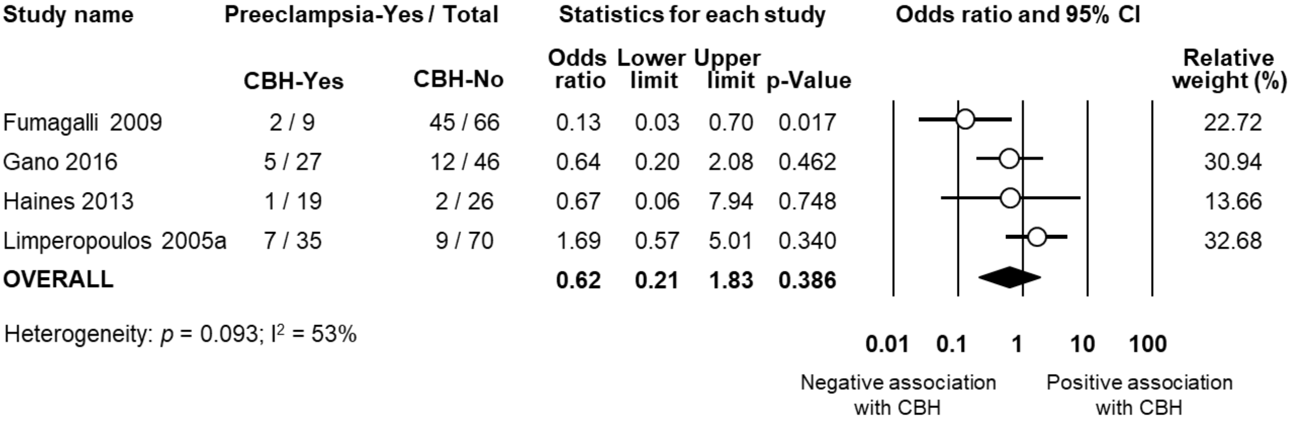

Supplementary Figure 3. Meta-analysis of cerebellar hemorrhage (CBH) and preeclampsia. CI: confidence interval.

CBH and antenatal steroids (k = 9)

Study name	ANS-Yes / Total: CBH-Yes	CBH-No	Odds ratio	Lower limit	Upper limit	p-Value	Relative weight
Biran 2011	6 / 10	18 / 20	0.17	0.02	1.15	0.069	5.56
Neubauer 2017	17 / 24	211 / 226	0.17	0.06	0.48	0.001	13.25
Gano 2016	22 / 27	41 / 46	0.54	0.14	2.06	0.364	9.54
Zayek 2012	51 / 79	770 / 1041	0.64	0.40	1.04	0.070	22.89
Steggerda 2013	11 / 16	70 / 92	0.69	0.22	2.21	0.533	11.49
Kidokoro 2014			0.70	0.26	1.86	0.474	13.95
Haines 2013	15 / 19	18 / 26	1.67	0.42	6.64	0.469	9.18
Limperopoulos 2005a	32 / 35	59 / 70	1.99	0.52	7.65	0.317	9.50
Fumagalli 2009	8 / 9	52 / 66	2.15	0.25	18.69	0.487	4.63
**OVERALL**			**0.65**	**0.39**	**1.07**	**0.091**	

Heterogeneity: *p* = 0.081; I^2 = 43%

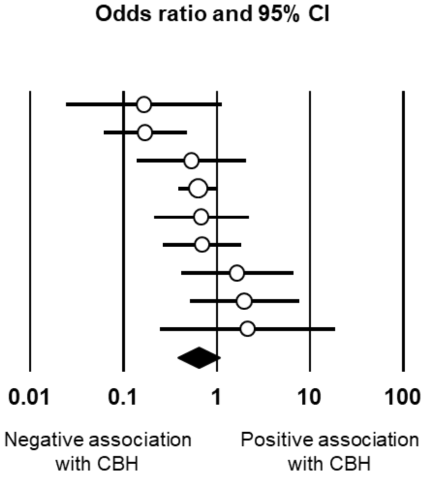

Supplementary Figure 4. Meta-analysis of cerebellar hemorrhage (CBH) and antenatal steroids. CI: confidence interval.

CBH and placental abruption (k = 3)

Study name	Placental abruption-Yes / Total: CBH-Yes	CBH-No	Odds ratio	Lower limit	Upper limit	p-Value	Relative weight (%)
Gano 2016	5 / 27	7 / 46	1.27	0.36	4.47	0.714	30.53
Limperopoulos 2005a	7 / 35	10 / 70	1.50	0.52	4.35	0.456	42.80
Haines 2013	7 / 19	5 / 26	2.45	0.64	9.44	0.193	26.67
**OVERALL**			**1.62**	**0.81**	**3.26**	**0.173**	

Statistics for each study; Odds ratio and 95% CI

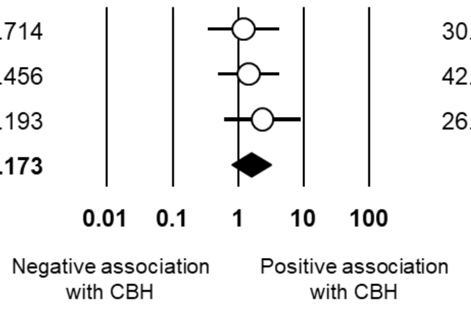

Heterogeneity: $p = 0.768$; $I^2 = 0\%$

Supplementary Figure 5. Meta-analysis of cerebellar hemorrhage (CBH) and placental abruption. CI: confidence interval.

CBH and chorioamnionitis (k = 5)

Study name	CA-Yes / Total: CBH-Yes	CBH-No	Odds ratio	Lower limit	Upper limit	p-Value	Relative weight
Kidokoro 2014			1.00	0.42	2.36	1.000	31.46
Haines 2013	6 / 19	8 / 26	1.04	0.29	3.72	0.954	14.27
Gano 2016	5 / 27	7 / 46	1.27	0.36	4.47	0.714	14.62
Limperopoulos 2005a	16 / 35	27 / 70	1.34	0.59	3.05	0.483	34.51
Biran 2011	2 / 10	2 / 20	2.25	0.27	18.93	0.455	5.13
**OVERALL**			**1.20**	**0.74**	**1.94**	**0.458**	

Statistics for each study; Odds ratio and 95% CI

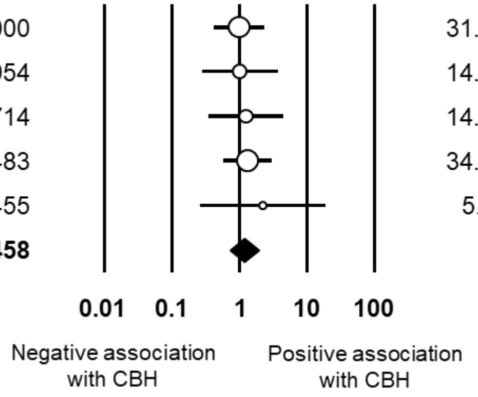

Heterogeneity: $p = 0.959$; $I^2 = 0\%$

Supplementary Figure 6. Meta-analysis of cerebellar hemorrhage (CBH) and chorioamnionitis. CI: confidence interval.

CBH and cesarean section (k = 9)

Study name	C-section-Yes / Total: CBH-Yes	C-section-Yes / Total: CBH-No	Statistics for each study: Odds ratio	Lower limit	Upper limit	p-Value	Relative weight
Neubauer 2017	19 / 24	213 / 226	0.23	0.07	0.72	0.011	10.82
Haines 2013	8 / 19	18 / 26	0.32	0.09	1.11	0.073	10.13
Fumagalli 2009	7 / 9	58 / 66	0.48	0.09	2.74	0.411	7.22
Zayek 2012	44 / 79	697 / 1041	0.62	0.39	0.99	0.043	15.64
Kidokoro 2014			1.10	0.47	2.59	0.827	12.87
Biran 2011	6 / 10	10 / 20	1.50	0.32	6.99	0.606	8.25
Steggerda 2013	11 / 16	54 / 92	1.55	0.50	4.82	0.451	10.80
Gano 2016	20 / 27	28 / 46	1.84	0.65	5.22	0.254	11.46
Limperopoulos 2005a	21 / 35	18 / 70	4.33	1.83	10.27	0.001	12.81
**OVERALL**			**0.95**	**0.52**	**1.76**	**0.875**	

Heterogeneity: $p < 0.001$; $I^2 = 71\%$

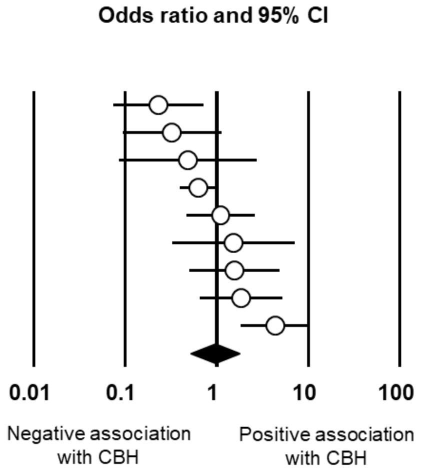

Supplementary Figure 7. Meta-analysis of cerebellar hemorrhage (CBH) and cesarean section. CI: confidence interval.

CBH and maternal age (mean difference in years; k = 3)

Study name	Sample size: CBH-Yes	Sample size: CBH-No	Statistics for each study: Difference in means	Lower limit	Upper limit	p-Value	Relative weight (%)
Fumagalli 2009	9	66	-1.60	-5.96	2.76	0.472	22.56
Haines 2013	19	26	-1.44	-5.03	2.15	0.432	33.25
Gano 2016	27	46	0.90	-2.21	4.01	0.571	44.20
**OVERALL**			-0.44	-2.51	1.63	0.675	

Heterogeneity: $p = 0.526$; $I^2 = 0\%$

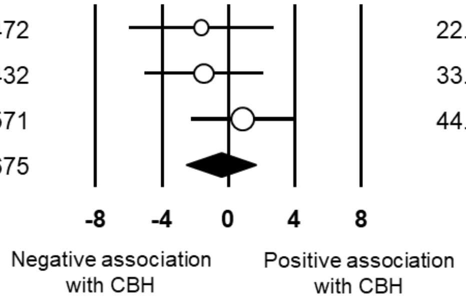

Supplementary Figure 8. Meta-analysis of cerebellar hemorrhage (CBH) and maternal age.

CBH and intubation (k = 6)

Study name	Intubation-Yes / Total CBH-Yes	CBH-No	Odds ratio	Lower limit	Upper limit	p-Value	Relative weight
Limperopoulos 2005a	32 / 35	58 / 70	2.21	0.58	8.40	0.246	11.11
Haines 2013	19 / 19	25 / 26	2.29	0.09	59.43	0.617	1.87
Zayek 2012	67 / 79	718 / 1041	2.51	1.34	4.71	0.004	50.29
Duerden 2013	17 / 21	70 / 112	2.55	0.80	8.09	0.112	14.89
Gano 2016	18 / 27	15 / 46	4.13	1.51	11.35	0.006	19.45
Fumagalli 2009	9 / 9	38 / 66	14.06	0.79	251.75	0.072	2.39
**OVERALL**			**2.84**	**1.82**	**4.44**	**0.000**	

Heterogeneity: p = 0.843; I^2 = 0%

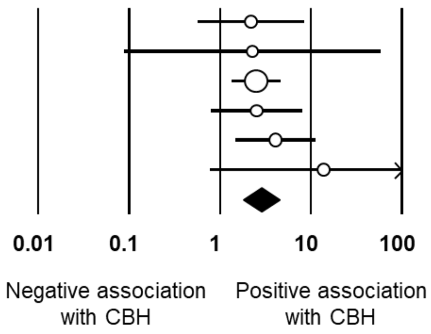

Supplementary Figure 9. Meta-analysis of cerebellar hemorrhage (CBH) and intubation. CI: confidence interval.

CBH and hypotension (k = 7)

Study name	Hypotension-Yes / Total CBH-Yes	CBH-No	Odds ratio	Lower limit	Upper limit	p-Value	Relative weight (%)
Steggerda 2013	7 / 16	23 / 92	2.33	0.78	6.97	0.129	10.82
Gano 2016	21 / 27	21 / 46	4.17	1.42	12.23	0.009	11.18
Limperopoulos 2005a	33 / 35	54 / 70	4.89	1.06	22.63	0.042	5.52
Zayek 2012	63 / 79	385 / 1041	6.71	3.82	11.78	0.000	40.92
Fumagalli 2009	5 / 9	10 / 66	7.00	1.60	30.66	0.010	5.94
Neubauer 2017	6 / 24	10 / 226	7.20	2.35	22.08	0.001	10.33
Kidokoro 2014			8.00	3.18	20.10	0.000	15.28
**OVERALL**			**5.78**	**4.04**	**8.29**	**0.000**	

Heterogeneity: p = 0.677; I^2 = 0%

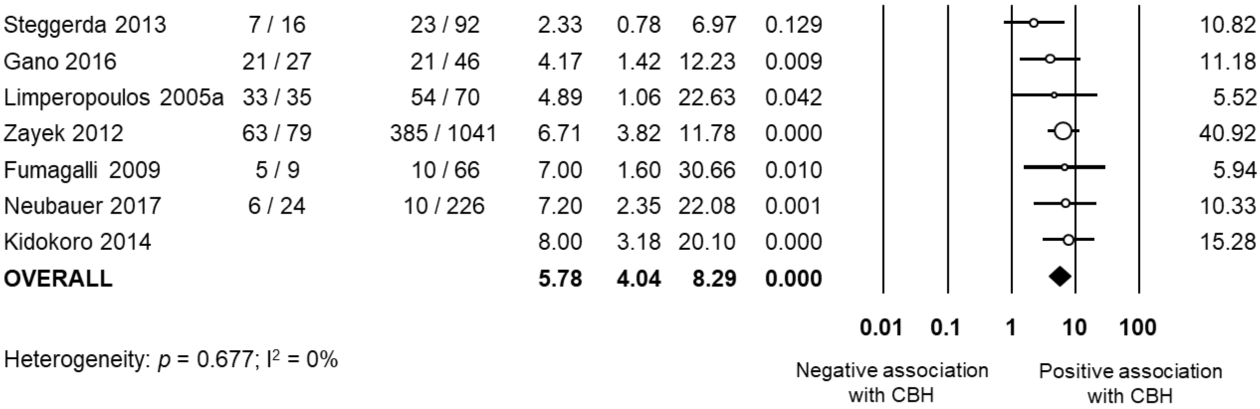

Supplementary Figure 10. Meta-analysis of cerebellar hemorrhage (CBH) and hypotension. CI: confidence interval.

CBH and PDA (k = 8)

Study name	PDA-Yes / Total: CBH-Yes	PDA-Yes / Total: CBH-No	Odds ratio	Lower limit	Upper limit	p-Value	Relative weight
Fumagalli 2009	6 / 9	43 / 66	1.07	0.24	4.68	0.929	6.40
Biran 2011	4 / 10	6 / 20	1.56	0.32	7.60	0.585	5.54
Steggerda 2013	5 / 16	20 / 92	1.64	0.51	5.26	0.408	10.23
Neubauer 2017	11 / 24	75 / 226	1.70	0.73	3.98	0.219	19.33
Haines 2013	11 / 19	11 / 26	1.88	0.57	6.21	0.304	9.72
Kidokoro 2014			2.90	1.30	6.48	0.010	21.53
Gano 2016	12 / 27	8 / 46	3.80	1.30	11.14	0.015	12.05
Limperopoulos 2005a	28 / 35	27 / 70	6.37	2.44	16.60	0.000	15.20
**OVERALL**			**2.48**	**1.68**	**3.67**	**0.000**	

Odds ratio and 95% CI

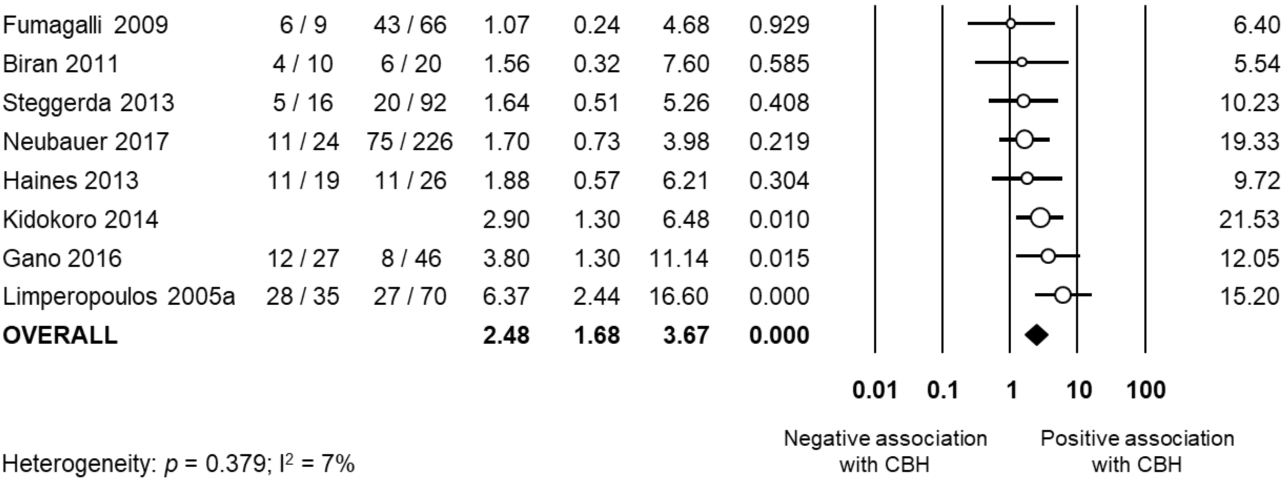

Heterogeneity: $p = 0.379$; $I^2 = 7\%$

Supplementary Figure 11. Meta-analysis of cerebellar hemorrhage (CBH) and patent ductus arteriosus (PDA). CI: confidence interval.

CBH and NEC (k = 6)

Study name	NEC-Yes / Total: CBH-Yes	NEC-Yes / Total: CBH-No	Odds ratio	Lower limit	Upper limit	p-Value	Relative weight
Gano 2016	0 / 27	3 / 46	0.23	0.01	4.55	0.331	4.11
Fumagalli 2009	0 / 9	3 / 66	0.95	0.05	19.97	0.976	4.00
Kidokoro 2014			2.80	0.79	9.96	0.112	22.99
Neubauer 2017	2 / 24	7 / 226	2.84	0.56	14.54	0.209	13.91
Steggerda 2013	1 / 16	2 / 92	3.00	0.26	35.18	0.382	6.11
Limperopoulos 2005a	22 / 35	18 / 70	4.89	2.05	11.67	0.000	48.88
**OVERALL**			**3.20**	**1.74**	**5.87**	**0.000**	

Odds ratio and 95% CI

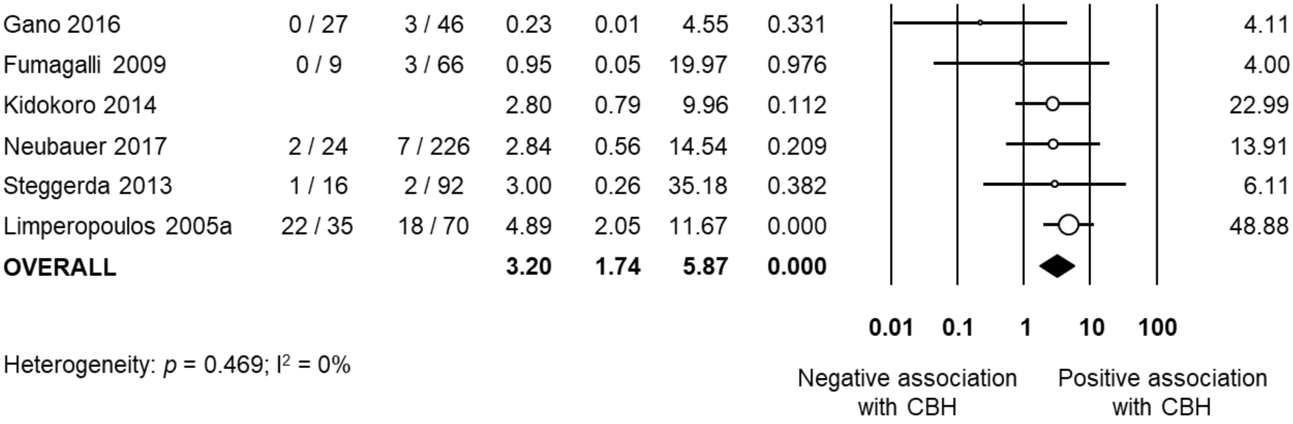

Heterogeneity: $p = 0.469$; $I^2 = 0\%$

Supplementary Figure 12. Meta-analysis of cerebellar hemorrhage (CBH) and necrotizing enterocolitis (NEC). CI: confidence interval.

CBH and sepsis (k = 10)

Study name	Sepsis-Yes / Total CBH-Yes	Sepsis-Yes / Total CBH-No	Statistics for each study: Odds ratio	Lower limit	Upper limit	p-Value	Relative weight (%)
Steggerda 2013	4 / 16	38 / 92	0.47	0.14	1.58	0.224	5.88
Fumagalli 2009	3 / 9	19 / 66	1.24	0.28	5.46	0.779	3.87
Kidokoro 2014			1.30	0.57	2.96	0.532	12.63
Gano 2016	12 / 27	16 / 46	1.50	0.57	3.96	0.413	9.04
Zayek 2012	40 / 79	385 / 1041	1.75	1.10	2.76	0.017	40.60
Limperopoulos 2005a	26 / 35	43 / 70	1.81	0.74	4.45	0.194	10.59
Biran 2011	5 / 10	6 / 20	2.33	0.49	11.17	0.289	3.48
Neubauer 2017	5 / 24	20 / 226	2.71	0.91	8.04	0.072	7.23
Haines 2013	17 / 19	15 / 26	6.23	1.19	32.75	0.031	3.10
Chau 2012	12 / 14	39 / 103	9.85	2.09	46.34	0.004	3.56
**OVERALL**			**1.81**	**1.22**	**2.68**	**0.003**	

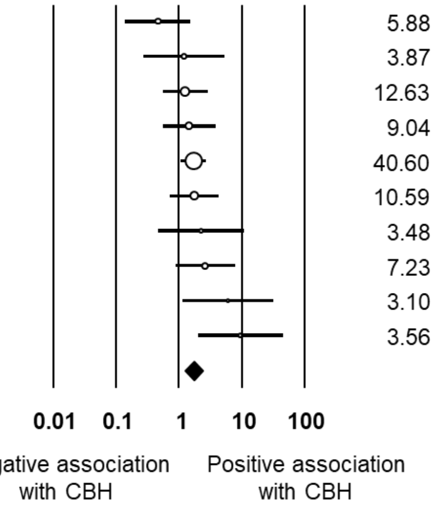

Heterogeneity: *p* = 0.158; I^2 = 31%

Supplementary Figure 13. Meta-analysis of cerebellar hemorrhage (CBH) and sepsis. CI: confidence interval.

CBH and BPD (k = 4)

Study name	BPD-Yes / Total CBH-Yes	BPD-Yes / Total CBH-No	Statistics for each study: Odds ratio	Lower limit	Upper limit	p-Value	Relative Weight (%)
Haines 2013	5 / 19	8 / 26	0.80	0.22	3.00	0.745	18.72
Gano 2016	10 / 27	11 / 46	1.87	0.67	5.26	0.235	25.28
Limperopoulos 2005a	25 / 35	37 / 70	2.23	0.93	5.33	0.071	30.22
Neubauer 2017	7 / 24	16 / 226	5.40	1.96	14.93	0.001	25.77
**OVERALL**			**2.21**	**1.11**	**4.43**	**0.025**	

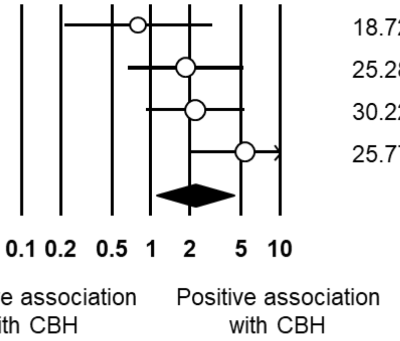

Heterogeneity: *p* = 0.150; I^2 = 44%

Supplementary Figure 14. Meta-analysis of cerebellar hemorrhage (CBH) and bronchopulmonary dysplasia (BPD). CI: confidence interval.

CBH and IVH grade 1-2 (k = 5)

Study name	IVH grade 1-2 / Total		Statistics for each study				Relative weight (%)
	CBH-Yes	CBH-No	Odds ratio	Lower limit	Upper limit	p-Value	
Tam 2011	3 / 9	29 / 85	0.97	0.22	4.14	0.962	7.00
Steggerda 2013	4 / 16	16 / 92	1.58	0.45	5.55	0.472	9.46
Gano 2016	7 / 27	8 / 46	1.66	0.53	5.25	0.386	11.24
Zayek 2012	39 / 79	239 / 1041	3.27	2.06	5.20	0.000	68.97
Fumagalli 2009	2 / 9	2 / 66	9.14	1.11	75.38	0.040	3.34
**OVERALL**			**2.41**	**1.40**	**4.13**	**0.001**	

Heterogeneity: $p = 0.264$; $I^2 = 24\%$

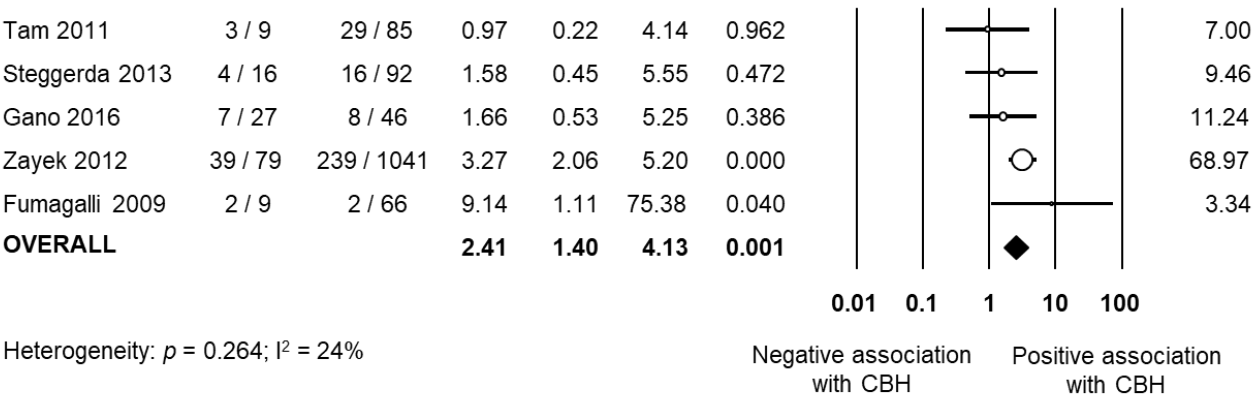

Supplementary Figure 15. Meta-analysis of cerebellar hemorrhage (CBH) and intraventricular hemorrhage (IVH) grade 1-2. CI: confidence interval.

CBH and IVH grade 3-4 (k = 6)

Study name	IVH grade 3-4-Yes / Total		Statistics for each study				Relative weight
	CBH-Yes	CBH-No	Odds ratio	Lower limit	Upper limit	p-Value	
Tam 2011	1 / 9	8 / 85	1.20	0.13	10.89	0.869	3.62
Gano 2016	4 / 27	3 / 46	2.49	0.51	12.11	0.257	7.03
Haines 2013	12 / 19	10 / 26	2.74	0.81	9.31	0.106	11.76
Fumagalli 2009	2 / 9	6 / 66	2.86	0.48	16.97	0.248	5.53
Zayek 2012	23 / 79	125 / 1041	3.01	1.79	5.06	0.000	64.88
Steggerda 2013	5 / 16	3 / 92	13.48	2.83	64.33	0.001	7.19
**OVERALL**			**3.16**	**2.08**	**4.80**	**0.000**	

Heterogeneity: $p = 0.516$; $I^2 = 0\%$

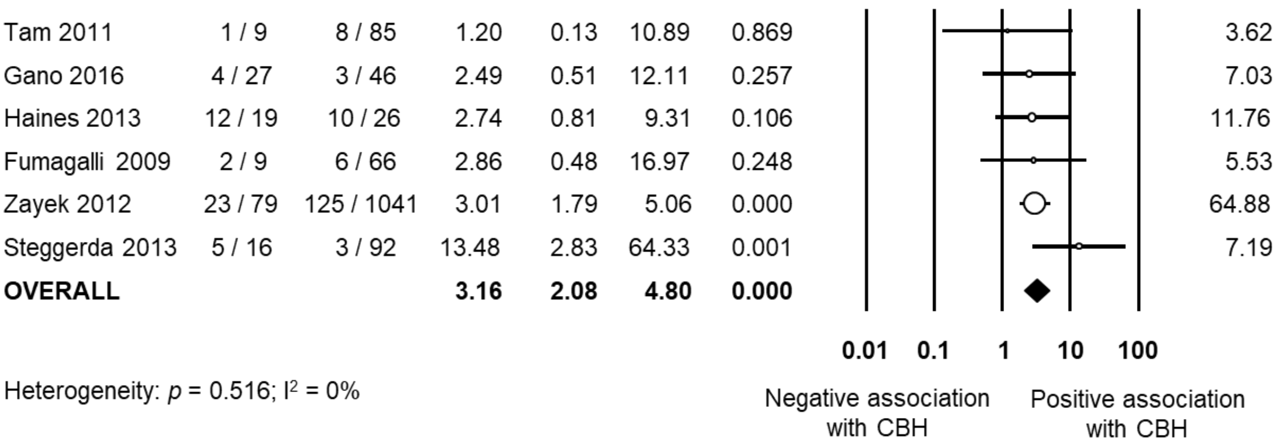

Supplementary Figure 16. Meta-analysis of cerebellar hemorrhage (CBH) and intraventricular hemorrhage (IVH) grade 3-4. CI: confidence interval.

3 References

Biran, V., Bodiou, A.-M., Zana, E., Gaudin, A., Farnoux, C., Hovhannisyan, S., Alison, M., Elmaleh, M., Oury, J.-F., and Maury, L. (2011). Lésions acquises du cervelet chez le grand prématuré: prévalence, facteurs de risque et conséquences fonctionnelles. Arch Pediatr 18, 261-266.

Chau, V., Brant, R., Poskitt, K.J., Tam, E.W., Synnes, A., and Miller, S.P. (2012). Postnatal infection is associated with widespread abnormalities of brain development in premature newborns. Pediatr Res 71, 274.

Duerden, E.G., Brown-Lum, M., Chau, V., Poskitt, K.J., Grunau, R.E., Synnes, A., and Miller, S.P. (2013). Resuscitation intensity at birth is associated with changes in brain metabolic development in preterm neonates. Neuroradiology 55, 47-54.

Dyet, L.E., Kennea, N., Counsell, S.J., Maalouf, E.F., Ajayi-Obe, M., Duggan, P.J., Harrison, M., Allsop, J.M., Hajnal, J., and Herlihy, A.H. (2006). Natural history of brain lesions in extremely preterm infants studied with serial magnetic resonance imaging from birth and neurodevelopmental assessment. Pediatrics 118, 536-548.

Fumagalli, M., Ramenghi, L.A., Righini, A., Groppo, M., Bassi, L., De Carli, A., Parazzini, C., Triulzi, F., and Mosca, F. (2009). Cerebellar haemorrhages and pons development in extremely low birth weight infants. Front Biosci 1, 537-541.

Gano, D., Ho, M.-L., Partridge, J.C., Glass, H.C., Xu, D., Barkovich, A.J., and Ferriero, D.M. (2016). Antenatal exposure to magnesium sulfate is associated with reduced cerebellar hemorrhage in preterm newborns. J Pediatr 178, 68-74.

Haines, K.M., Wang, W., and Pierson, C.R. (2013). Cerebellar hemorrhagic injury in premature infants occurs during a vulnerable developmental period and is associated with wider neuropathology. Acta Neuropathol Commun 1, 69.

Kidokoro, H., Anderson, P.J., Doyle, L.W., Woodward, L.J., Neil, J.J., and Inder, T.E. (2014). Brain injury and altered brain growth in preterm infants: predictors and prognosis. Pediatrics, peds. 2013-2336.

Limperopoulos, C., Bassan, H., Gauvreau, K., Robertson, R.L., Sullivan, N.R., Benson, C.B., Avery, L., Stewart, J., Soul, J.S., and Ringer, S.A. (2007). Does cerebellar injury in premature infants contribute to the high prevalence of long-term cognitive, learning, and behavioral disability in survivors? Pediatrics 120, 584-593.

Limperopoulos, C., Benson, C.B., Bassan, H., Disalvo, D.N., Kinnamon, D.D., Moore, M., Ringer, S.A., Volpe, J.J., and Du Plessis, A.J. (2005). Cerebellar hemorrhage in the preterm infant: ultrasonographic findings and risk factors. Pediatrics 116, 717-724.

Neubauer, V., Djurdjevic, T., Griesmaier, E., Biermayr, M., Gizewski, E.R., and Kiechl-Kohlendorfer, U. (2017). Routine magnetic resonance imaging at term-equivalent age detects brain injury in 25% of a contemporary cohort of very preterm infants. PloS one 12, e0169442.

O'shea, T.M., Kuban, K.C., Allred, E.N., Paneth, N., Pagano, M., Dammann, O., Bostic, L., Brooklier, K., Butler, S., and Goldstein, D.J. (2008). Neonatal cranial ultrasound lesions and developmental delays at 2 years of age among extremely low gestational age children. Pediatrics 122, e662.

Steggerda, S.J., De Bruïne, F.T., Van Den Berg-Huysmans, A.A., Rijken, M., Leijser, L.M., Walther, F.J., and Van Wezel-Meijler, G. (2013). Small cerebellar hemorrhage in preterm infants: perinatal and postnatal factors and outcome. Cerebellum 12, 794-801.

Tam, E.W., Rosenbluth, G., Rogers, E.E., Ferriero, D.M., Glidden, D., Goldstein, R.B., Glass, H.C., Piecuch, R.E., and Barkovich, A.J. (2011). Cerebellar hemorrhage on magnetic resonance imaging in preterm newborns associated with abnormal neurologic outcome. J Pediatr 158, 245-250.

Zayek, M., Benjamin, J., Maertens, P., Trimm, R., Lal, C., and Eyal, F. (2012). Cerebellar hemorrhage: a major morbidity in extremely preterm infants. J Perinatol 32, 699.
